# Tannic acid breaks hepatic persistence of *Salmonella* and promotes Kupffer cell M2 polarization via the gut-liver axis in broilers

**DOI:** 10.1128/spectrum.03188-25

**Published:** 2026-09-21

**Authors:** Fangrui Guo, Yinzhu Zhang, Aoao Wu, Liu Xu, Xiangyan Liu, Yuanyuan Zhu, Bo Xiao, Xiaohong Xie, Qian He, Yanbin Ding, Chuan Zhou, Zhihang Yuan, Jinhui Liu, Lixin Wen, Ji Wang

**Affiliations:** 1 Hunan Engineering Research Center of Livestock and Poultry Health Care, College of Veterinary Medicine, Hunan Agricultural University 12575, Changsha, China; 2 Changsha Lvye Biotechnology Co., Ltd., Changsha, China; 3 Yuelushan Laboratory, Changsha, China; 4 Hunan Collaborative Innovation Center of Animal Production Safety, Changsha, China; The University of Tennessee Southern, Pulaski, Tennessee, USA

**Keywords:** *Salmonella*, tannic acid, gut-liver axis, Kupffer cells, broilers

## Abstract

*Salmonella* is a significant foodborne threat to the poultry industry, particularly affecting chicks. Tannic acid (TA), a polyphenol found in plants like chestnut and plum trees, offers antioxidant and anti-inflammatory benefits. This study examined the protective effects of TA on growth performance, gut microbiota, gut barrier function, and liver immune damage in broilers infected with *Salmonella*. Results indicated that *Salmonella* infection significantly reduced chick growth (*P* < 0.05) and compromised intestinal health, leading to chronic inflammation. Although *Salmonella* was undetectable in the intestines and feces 10 days post-infection, it translocated to the liver, causing subclinical infections. This resulted in M1 polarization of hepatic Kupffer cells and increased inflammatory factors (*P* < 0.05), disrupting liver immune homeostasis. Dietary supplementation with 200 mg/kg TA could effectively improve their growth performance, protect the intestinal barrier, promote intestinal health, inhibit the colonization of *Salmonella* in the liver, repair the immune microenvironmental homeostasis in the liver, and improve the hepatic immune function of the broilers. This study suggests that TA protects the intestines and liver from *Salmonella* damage by reducing inflammation, repairing the intestinal barrier, decreasing *Salmonella* colonization in the liver, improving the liver’s immune environment, and modulating metabolic disorders. This study elucidates the mechanism by which *Salmonella* induces persistent liver colonization and hepatic immune dysregulation through the gut-liver axis. Furthermore, it highlights TA’s potential as an antibiotic alternative to reduce *Salmonella*-induced liver immune damage through the gut-liver axis, offering a new approach to poultry health management.

**IMPORTANCE:**

This research addresses a critical challenge in poultry production: controlling persistent *Salmonella* infections that evade conventional treatments and contribute to foodborne illness. We show how *Salmonella* can “hide” in the liver after disappearing from the gut, causing ongoing health problems for the birds. More importantly, our findings reveal that a natural plant compound, tannic acid, can effectively break this cycle. Instead of directly killing the bacteria, it works by strengthening the gut and recalibrating the liver’s immune defenses. This offers a novel, host-directed strategy to combat bacterial infections. Our work provides a scientific foundation for using tannic acid as a sustainable antibiotic alternative in animal feed, which could lead to healthier poultry and safer food products, with significant implications for both animal agriculture and public health.

## INTRODUCTION

As zoonotic enterobacteria, *Salmonella* species are capable of causing various forms of salmonellosis, including gastroenteritis and septicemia, posing significant threats to public health and food safety (1, 2). The global poultry industry has experienced substantial growth due to the increasing consumer demand for chicken meat and eggs. However, *Salmonella* predominantly infects chicks and is associated with a high mortality rate. It can stick to intestinal epithelial cells, leading to inflammation, injury, barrier dysfunction, immune damage, reduced growth, and diarrhea (3–5). In recent years, decreased antimicrobial sensitivity and increased resistance were attributed to the long-term antibiotic treatment in broilers (6). Thus, natural, safe, and efficient substitutes are crucial for preventing *Salmonella* in chicks and reducing economic losses in the poultry industry.

The liver is a crucial organ in vertebrates, playing a central role in metabolism and various physiological functions. However, its importance as a major immune organ, characterized by a high density of myeloid and lymphoid immune cells, is often underestimated (7, 8). Kupffer cells, the liver-resident macrophages, are integral to the liver’s immune function. The homeostasis of Kupffer cells is responsible for clearing pathogens and damaged red blood cells from the intestine, regulating iron and fat metabolism, and maintaining immune tolerance (9). Notably, the liver hosts a dynamic microbiome, and liver immunity is fundamentally dependent on this microbial community (10). Disruptions in gut microbiota, such as those caused by *Salmonella* infection, can compromise intestinal barrier function. This disruption results in a decrease in intestinal flora metabolites, which impairs the transformation of hepatic Kupffer cells into their anti-inflammatory phenotype (11). Concurrently, the compromised barrier permits microbial components to translocate to the liver, triggering inflammation and immune responses (11). Collectively, the liver’s immune cells, including Kupffer cells, its resident microbiota, and the associated metabolites, constitute the liver immune microenvironment.

Tannic acid (TA), a polyphenolic compound mainly found in the bark and fruit of plants like chestnut and plum trees, has antioxidant and anti-inflammatory properties, making it a valuable and safe food additive (12). Previous studies have demonstrated that TA possesses a variety of biological functions, including neuroprotective effects (13), enhancement of intestinal development (14), and inhibition of mold, fungal, and bacterial infections (15–18). Tannins have astringent, antimicrobial, and antioxidant effects in the gut (19). These effects may indirectly affect the composition and activity of the gut microbiota, which interacts closely with the liver microbiota. Consequently, this study primarily investigated the impact and underlying mechanisms of TA on hepatic immune damage induced by *Salmonella* in broilers. The findings aim to enhance the understanding of *Salmonella*-induced dysbiosis in broilers and propose novel strategies for improving livestock production.

## MATERIALS AND METHODS

### Animal treatment and experimental design

*Salmonella* (CICC 21510) was purchased from the China Industrial Microbial Strain Preservation and Management Center, and chestnut tannins (≥70%) were purchased from Guangdong Putland Biotechnology Co. The 1-day-old AA broilers were used for the experiment, and the test animals were kept in a controlled environment (light/dark cycle for 12 hours, constant humidity, and constant temperature) with free access to water and broiler basal ration (the test ration was prepared with reference to the Chinese broiler feeding standard [NY/T 33-2004], and its composition and nutritional level are shown in Table 1). Based on our team’s previous study (20), which concluded that 200 mg/kg of chestnut tannins from different sources had the most significant effect on broiler growth performance, 200 mg/kg was chosen as the low dose of tannins for this study.

**TABLE 1:** Dietary composition and nutritional level of the experimental broilers (%)

Item (%)	Content
Diet composition	
Corn	54.00
Soybean meal	38.00
Soybean oil	4.00
Calcium phosphate	0.60
Stone flour	1.99
Salt	0.24
DL-methionine	0.11
L-lysine	0.08
Choline chloride	0.10
Mineral premix^*a*^	0.86
Vitamin premix^*b*^	0.02
Total	100
Nutrient level	
Metabolic energy (MJ/kg）	12.65
Crude protein (%）	21.09
Calcium (%）	1.00
Phosphorus (%）	0.45

^*a*^ Premix provides for minerals per kg of feed: zinc, 75.00 mg; copper, 5.30 mg; iron, 114.60 mg; manganese, 121.40 mg; and selenium, 0.09 mg.

^*b*^ Premix provides for vitamins per kg of feed: vitamin A, 8,870 IU; vitamin D, 2,840 IU; vitamin E, 23.6 IU; vitamin K, 2.36 mg; vitamin K2, 2.36 mg; D-pantothenic acid, 2.36 mg; biotin, 0.03 mg. 2.36 mg; vitamin B1, 2.36 mg; vitamin B2, 7.10 mg; vitamin B3, 0.33 mg; vitamin B12, 0.03 mg; biotin, 0.12 mg; niacinamide, 37.82 mg; and folic acid, 1.18 mg.

Two hundred fifty healthy 1-day-old white-feathered broilers of approximately the same body weight were selected as test animals and randomly divided into five groups with five replicates of 10 chickens each, and the test period was 21 days, which were divided into a control group (CON), a *Salmonella*-infected group (SALM, 3 × 10^8^ CFU/mL) (21), a low-dose group of TA (STL, 3 × 10^8^ CFU/mL + TA, 200 mg/kg), a high-dose group of TA (STH, 3 × 10^8^ CFU/mL + TA, 400 mg/kg), and TA high-dose fed alone group (TH, 400 mg/kg). Broilers were challenged on day 10 of age, and the bacterial concentration was adjusted to 3 × 10^8^ CFU/mL with sterile phosphate-buffered saline (PBS). Then, 1 mL of *Salmonella* solution was administered via gavage to each chicken in the SALM, STL, and STH groups for two consecutive days. In contrast, 1 mL of sterile PBS was gavaged to the CON and TH groups, following the same treatment schedule and handling procedures used for the tannic *Salmonella*-exposed groups. This vehicle control protocol ensured that the observed effects could be attributed specifically to TA rather than stress from gavage manipulation or solvent exposure. Samples were collected after the chickens reached 21 days of age. Details of TA dose, infection and treatment times, and the specific assays used for each tissue are provided in Table 1.

### Sample collection

Broilers were weighed and labeled prior to blood collection at 8 a.m. on day 21 of feeding. Blood was collected from the inferior iliac vein, then the animal was euthanized and dissected to obtain tissue samples. The collected blood is allowed to settle overnight in a refrigerator at 4°C. Serum was collected for subsequent testing. The liver tissue and jejunum of each broiler were weighed, and the jejunum and liver were divided into two parts. The left liver lobe was fixed with neutral formaldehyde, snap-frozen in liquid nitrogen, and stained with hematoxylin and eosin (H&E). The other part was immediately frozen in liquid nitrogen and then stored at −80°C for further analysis.

### Biochemical analysis

The alanine aminotransferase (ALT) and aspartate aminotransferase (AST) assays were performed according to the operating instructions of the Myriad Automatic Animal Serum Biochemistry Instrument (22).

### ELISA

Supernatants from centrifuged serum and homogenized liver tissue (~1 g) were analyzed. For homogenate preparation, liver tissue was homogenized in saline (1:9, wt/vol) and centrifuged (3,000 rpm for 10 min at 4°C). The resulting supernatant (10% homogenate) and serum were assayed directly. Concentrations of IgM, IgG (IgY), lysozyme, and lipopolysaccharide (LPS) were measured using ELISA kits (Shanghai Win-win Biotechnology Co., Ltd., China) according to the manufacturer’s instructions, with absorbance read on a multifunction microplate reader.

### Histopathological analysis (hematoxylin-eosin and periodic acid-Schiff stain)

Liver and jejunal tissue samples were dehydrated, cleared, embedded in paraffin, fixed in 10% neutral-buffered paraformaldehyde, and sectioned; the thickness of the sections was 5 mm, and H&E staining was performed using standard methods. Subsequently, morphological changes in the liver and adipose tissue were observed under a Nikon microscope. Paraffin sections were deparaffinized in water (cell smears and frozen sections were washed directly in water). Sections were rinsed in distilled water, treated with a clear solution of periodate for 10 min, rinsed in tap water for 10 min, incubated in Schiff’s solution for 10 min, rinsed in running water for 5 min, restained with Mayer’s hematoxylin for 2 min, rinsed in tap water until nuclei turned blue, rinsed in running water for 5 min, and finally dehydrated, cleared, and blocked (23). Image-Pro Plus 6 software (Media Cybernetics, Inc.) was used to calculate the number of cup cells in each group of samples from the jejunum. Liver inflammation scores were evaluated according to reference 24.

### Quantitative real-time PCR analysis

Liver and jejunal tissues were collected, quickly frozen in liquid nitrogen, and stored at −80°C for subsequent experiments. A high-throughput PCR instrument (WaferGen Biosystems) was utilized. Primer sequences are listed in Table 2, and negative controls were included. Specific methods can be derived from our previous research (25).

**TABLE 2:** Primer sequences for quantitative real-time PCR analysis

Gene	GenBank ID	Primer sequence (5′−3′)
*Claudin-1*	NM_001013611.2	F: GGGGACAACATCGTGACCG R: AGGAGTCGAAGACTTTGCACT
*MUC2*	XM_040673077.2	F: CAGGATACGTGTGTGCCCAT R: GGACGCGTTGCAATCAAAGT
*Occludin*	XM_046904540.1	F: CCTCATCGTCATCCTGCTCT R: GGTCCCAGTAGATGTTGGCT
*IL-6*	NM_204628	F: TCTGTTCGCCTTTCAGACCTA R: GACCACCTCATCGGGATTTAT
*IL-10*	NM_001004414.2	F: GCTGAGGGTGAAGTTTGAGGAA R: GAAGCGCAGCATCTCTGACA
*TNF-α*	NM_204267	F: AGATGGGAAGGGAATGAACC R: TCAGAGCATCAACGCAAAAG
*β-actin*	L08165	F: ATCCGGACCCTCCATTGTC R: AGCCATGCCAATCTCGTCTT
*iNOS*	NM_204961.1	F: TGGGTGGAAGCCGAAATA R: GTACCAGCCGTTGAAAGGAC
*IL-1β*	NM_204524.1	F: CATCACCAACCAACCCGA R: ACGAGATGGAAACCAGCAA
*ZO-1*	XM_046925214.1	F: TGGCAATCAACTTTGGGTAGCA R: ATCCACAGAGGCAACTGAACCATA
*IFN-γ*	NM_205149.1	F: TGAGCCAGATTGTTTCGATG R: CTTGGCCAGGTCCATGATA
*CCL1*	NM_204776.2	F: CCAGCAGCACCAAACCAGCA R: ACTTGCCCGAGCTCTCAGTCC
*CCL4*	NM_204720.3	F: AGACCGCCCCGCATCTGCCC R: GTCCCACTGGGGCAGAGGA
*IL-18*	XM_046932263.1	F: GCTGGAATGCGATGCCTTTT R: TGGACGAACCACAAGCAACT
*IL-1*	XM_015297469.3	F: CTCCTCCAGCCAGAAAGTGA R: GAGCTTGTAGCCCTTGATGC

### Immunofluorescence analysis

The protein expression in liver tissues was detected by immunofluorescence analysis as described previously (20). Protein immunofluorescence intensity was quantified using Image-Pro Plus 5.1 software (Media Cybernetics, Inc.). Immunofluorescent antibodies MUC2, Claudin-1, CD80, CD206, IL-1β, TNF-α, and NF-κB were purchased from Hunan Wonderwave Biotechnology Co., Ltd.

### Gut microbiota analysis

Microbial genomic DNA was extracted from the jejunal contents. The analysis of intestinal microbial diversity was commissioned to Majorbio Bio-Pharm Technology Co., Ltd. in Shanghai, China. Metagenomic sequencing data were analyzed on the free online Majorbio Cloud platform (26). Sequencing of the 16S rRNA gene targeting the V3-V4 hypervariable regions was performed on an Illumina NovaSeq platform. Operational taxonomic unit (OTU) clustering and chimera elimination were conducted using UPARSE software (v7.1) on quality-controlled, spliced sequences based on a 97% similarity threshold.

### Liver microbiota analysis

The liver microbiota diversity test was commissioned to Guangzhou Gene Denovo Biology Co., Ltd. (27).

### Liver tissue bacterial culture and enumeration

Liver tissues stored at −80°C were thawed on ice. Approximately 0.5 g of tissue was aseptically weighed and transferred into a 5 mL centrifuge tube containing 1 mL of sterile phosphate-buffered saline. Homogenization was performed on ice using a tissue homogenizer set to 65 Hz for 1 min, with the cycle repeated twice. The homogenate was then kept at 4°C for 30 min to allow settling. Subsequently, the supernatant was subjected to serial 10-fold dilutions in sterile PBS. From the 10^−5^ dilution, a 100 µL aliquot was spread onto both LB agar and MacConkey (MAC) agar plates using the glass bead spreading method. All plates were incubated at 37°C for 24 hours. Following incubation, colonies were counted, and the *Salmonella* concentration was calculated and expressed as colony-forming units per milliliter (CFU/mL).

### Isolation and identification of *Bacteroides*

Samples of jejunal contents stored at −80°C were processed in an anaerobic chamber. Serial dilutions were prepared in brain heart infusion (BHI) broth, plated onto Columbia blood agar and Bacteroides Bile Esculin agar, and incubated anaerobically at 37°C for 48 hours. Distinct single colonies from plates with 30–300 colonies were selected, streaked twice onto fresh Columbia blood agar for purification, and then inoculated into BHI broth for anaerobic enrichment at 37°C for 16–18 hours. Genomic DNA was extracted from the enriched culture. The near-full-length 16S rRNA gene was amplified by PCR using universal primers 27F (5′-AGAGTTTGATCCTGGCTCAG-3′) and 1492R (5′-TACGGCTACCTTGTTACGACTT-3′) (Sangon Biotech, Shanghai). The PCR reaction consisted of 25 μL PrimeSTAR Max Premix, 2.5 μL each of the forward and reverse primers (10 μmol/L), 1 μL of bacterial template, and 19 μL of water. The thermal cycling conditions were as follows: initial denaturation at 98°C for 10 s, followed by 35 cycles of 98°C for 10 s, 58°C for 5 s, and 72°C for 10 s. The PCR products were purified and sequenced bidirectionally by Tsingke Biotechnology using the amplification primers. The obtained consensus sequence was compared against the NCBI nucleotide database using BLASTN for species identification.

For phylogenetic tree construction, the obtained 16S rRNA gene sequence of the isolate was submitted to the NCBI database for BLAST homology analysis. The results, including similarity percentage, score, and sequence length, were used to select reference sequences. Typically, the top one or two sequences with the highest similarity, along with approximately 10–13 other sequences showing lower similarity, were chosen. These selected sequences, along with our isolate sequence, were compiled after downloading in FASTA format. An additional sequence from a distantly related genus within the same family was included as an outgroup. All sequences were aligned using the “Create a new alignment” function in MEGA11 software, and a phylogenetic tree was subsequently constructed.

### Co-culture of TA and anabolic bacteria

Bacteroides was isolated from broiler jejunal contents. For the co-culture experiment, 1 mL of the bacterial suspension (approximately 6.8 × 10^6^ CFU) was inoculated into BHI broth along with 1 mL of TA solution, resulting in a final concentration of 32 µg/mL TA in the culture system. The co-culture was then placed in an anaerobic bag to maintain an anaerobic environment and incubated statically at 37°C for 16–18 hours. Following incubation, the bacterial culture was processed, and the supernatants were analyzed using high-performance liquid chromatography to quantify the remaining TA and its metabolite, gallic acid.

### Statistical analyses

Data are presented as the mean ± standard error of the mean (SEM). All statistical analyses were performed using SPSS 25.0 (IBM Corp., Armonk, NY, USA). The normality of data distribution was verified using the Shapiro–Wilk test. Depending on the outcome of the normality test, parametric data were analyzed by one-way analysis of variance (ANOVA), and non-parametric data were analyzed using the Kruskal–Wallis *H* test. For *post hoc* comparisons following significant ANOVA results, Tukey’s test was applied; for the Kruskal–Wallis test, Dunn’s test with Bonferroni correction was used. This statistical approach was applied to all quantitative data, including broiler growth performance, serum biochemical indicators, microbial alpha diversity indices, relative taxonomic abundance, and results from ELISA, qPCR, and co-culture experiments. To control the family-wise error rate due to multiple comparisons (e.g., across TA dose groups or between treatment conditions), Bonferroni correction was employed in all *post hoc* analyses to calculate adjusted *P*-values, maintaining a conservative threshold of *α* = 0.05. Statistical significance was defined as *P* < 0.05, and *P* < 0.01 was considered highly significant (two-tailed tests).

## RESULTS

### Effects of TA on growth performance, biochemical indices, and intestinal function of broilers

**Fig 1:**
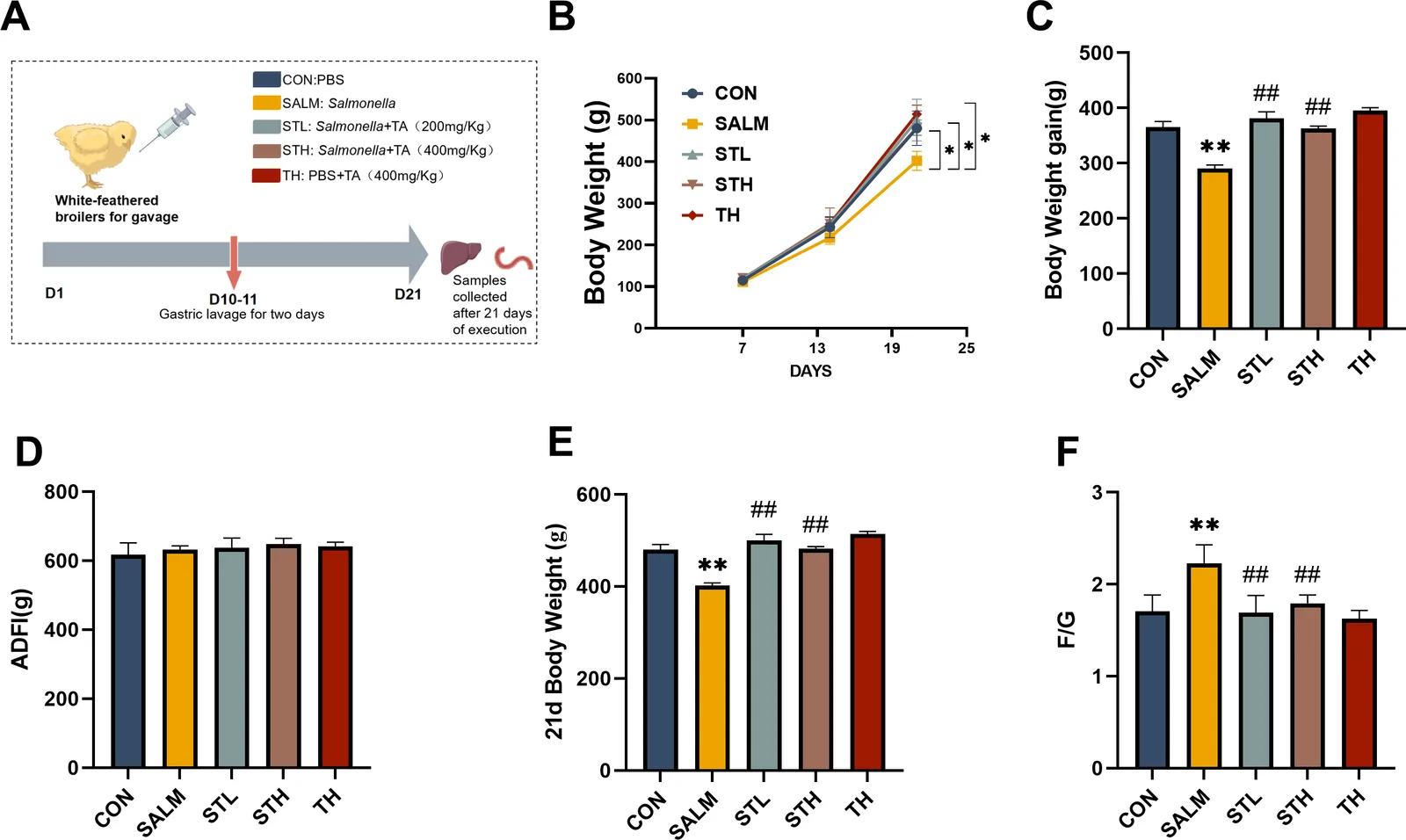
Tannic acid improves *Salmonella*-induced growth performance of broilers. (**A**) Experimental flow chart. (**B**) Body weight trend. (**C**) Body weight gain. (**D**) Average daily feed intake over 21 days. (**E**) Body weight at 21 day. (**F**)Ratio of total feed intake to weight gain. All data are expressed as mean ± SEM (*n* = 15). All means are analyzed by one-way ANOVA; * denotes the level of significance compared to the CON group; *P < 0.05 and **P < 0.01. # denotes significance compared to the SALM group, #*P* < 0.05 and ##*P* < 0.01.

The experimental flowchart is shown in Fig. 1A. There was no significant difference in body weight gain among all groups at 14 days of rearing (Fig. 1B) (*P* > 0.05). However, after 14 days, the body weight gain in the SALM group was significantly lower than that of the other groups (Fig. 1B). At the end of the experiment, the final body weights of broilers in the SALM group were also significantly lower than those in the CON, STL, and STH groups (*P* < 0.01, Fig. 1C). In contrast, there was no significant difference in the mean feed intake between the groups (*P* > 0.05, Fig. 1D). In addition, the daily weight gain of the SALM group was significantly lower than that of the CON, STL, and STH groups (*P* < 0.01, Fig. 1E), resulting in a significantly higher feed-to-meat ratio than the other groups (*P* < 0.01, Fig. 1F).

**Fig 2:**
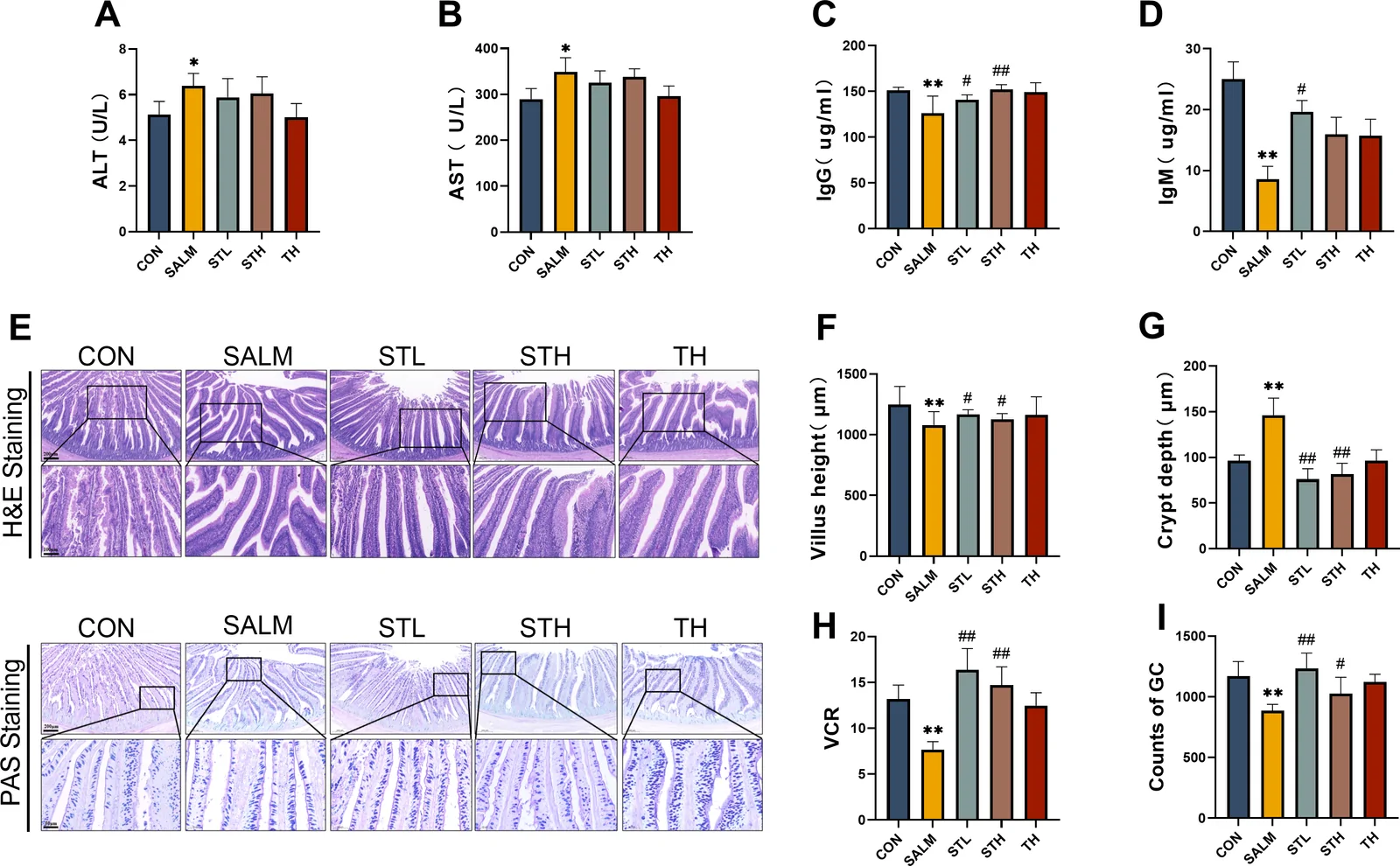
Tannic acid ameliorates *Salmonella*-induced biochemical disturbances and jejunal damage in broilers. (**A**) Serum ALT; (**B**) serum AST; (**C**) serum IgG; (**D**) serum IgM; (**E**) histopathological variation in jejunum tissues (H&E staining; ×100; scale bar = 200 µm/100 μm). Distribution of goblet cells in intestine was determined by PAS staining (×200; scale bar = 50 µm/200 μm). ; (**F**) villus length; (**G**) crypt depth; (**H**) VCR; (**I**) cup cell count; all data are shown as the mean ± SEM (*n* = 7). (**A–D**) *n* = 13; (**F–I**) *n* = 3, all means are analyzed by one-way ANOVA. * denotes the level of significance compared to the CON group, *P < 0.05 and **P < 0.01. # denotes significance compared to the SALM group, #*P* < 0.05 and ##*P* < 0.01.

As shown in Fig. 2, serum AST and ALT levels were not significantly higher in the SALM group compared with the CON, STL, and STH groups (*P* > 0.05, Fig. 2B). In addition, serum IgG and IgM levels were significantly lower in the SALM group compared with the CON, STL, and STH groups (*P* = 0.0013, *P* = 0.0007, and *P* = 0.0029, Fig. 2C and D). To evaluate intestinal changes after SALM exposure and the therapeutic effects of TA, H&E staining was used to identify histopathological changes, and PAS staining assessed the impact on the mucin layer and goblet cell count. Histopathological changes observed through H&E staining (Fig. 2E) revealed that the jejunal tissue of the control group exhibited normal morphology, clear boundaries, and neatly arranged epithelial cells. In contrast, damage to the jejunum caused by SALM was characterized by the disruption and curling of intestinal villi, along with a significant reduction in villus length. Compared to the control group, the depth of crypts was exacerbated, and the villus-to-crypt ratio (VCR) was significantly decreased in the SALM group (*P* < 0.0001); however, this damage was alleviated following TA treatment, particularly in the STL group (Fig. 2F and H) (*P* < 0.0001). Similar findings were observed with PAS staining. The intestinal mucosa of the control group was covered with a thick, continuous mucin layer containing numerous purple-blue goblet cells. Following exposure to *Salmonella*, the mucin layer became thinner, and the number of goblet cells was significantly reduced, although the STL and STH groups showed signs of recovery (*P* = 0.0028, *P* = 0.0003, and *P* = 0.0353) (Fig. 2I). These results indicate that *Salmonella* can cause significant damage to serum biochemistry and the jejunum.

### Effect of TA on *Salmonella*-induced liver in broilers

**Fig 3:**
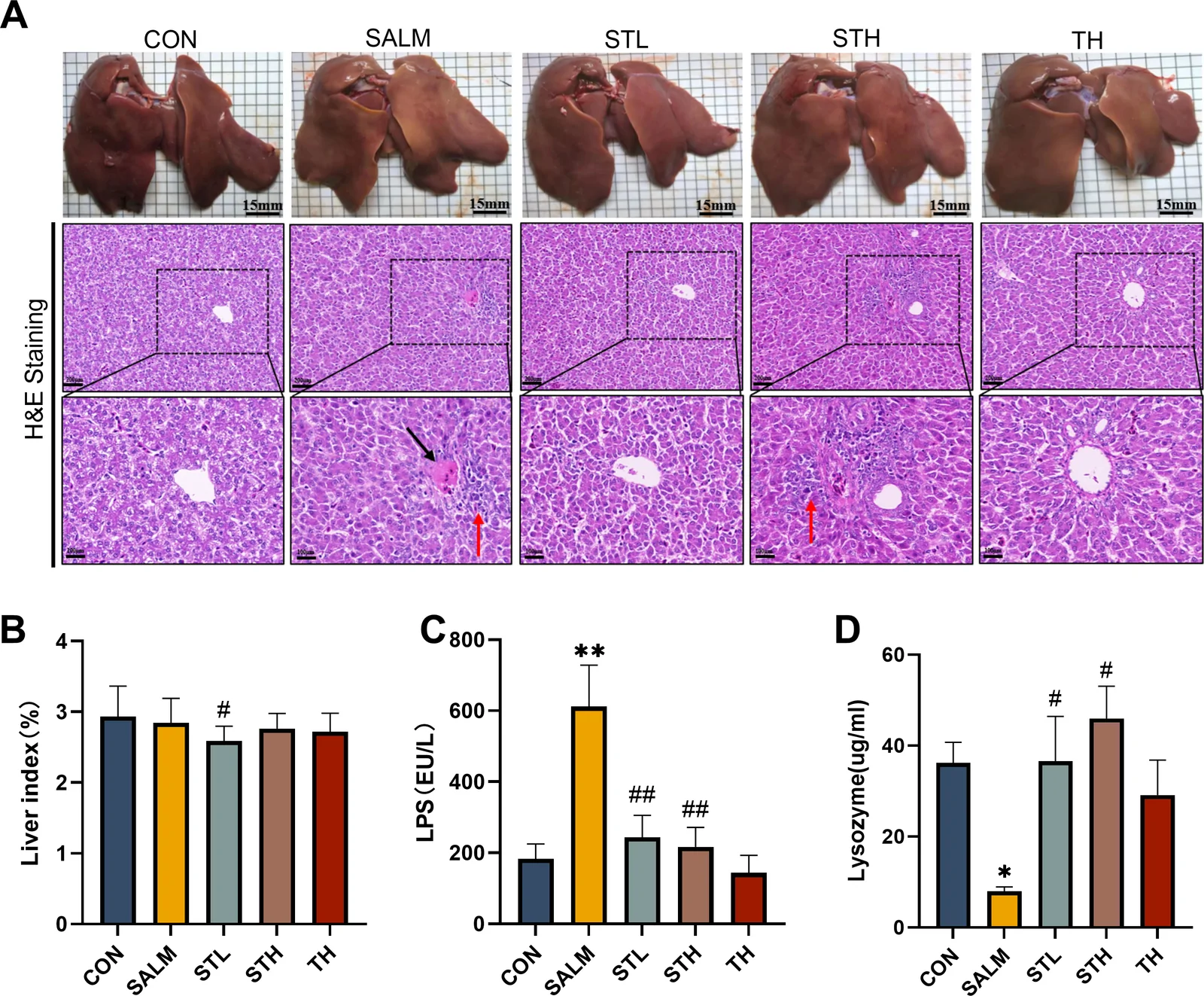
Effect of tannic acid on *Salmonella*-induced broiler liver. (**A**) Liver macrophotography, histopathological variation in liver tissues (×200/×400; scale bar = 200 µm/100 μm), inflammatory cell infiltration (red arrows), and erythrocyte sludge (black arrows); (**B**) liver index; (**C**) liver LPS; (**D**) liver lysozyme; all data are shown as the mean ± SEM (*n* = 7). *n* = 3, all means are analyzed by one-way ANOVA; * denotes the level of significance compared to the CON group, *P < 0.05 and **P < 0.01. # denotes significance compared to the SALM group, #*P* < 0.05 and ##*P* < 0.01.

As illustrated in Fig. 3, gross examination revealed that the liver surface exhibited a more pronounced yellow coloration in the SALM group. Correspondingly, histopathological findings indicated that the liver in the SALM group displayed inflammatory cell infiltration (red arrows) and portal erythrocyte stasis (black arrows) when compared to the control group (Fig. 3A). In addition, the liver index of the STH group was lower than that of the other groups, but the results were not significant (*P* > 0.05, Fig. 3B). The liver LPS levels in the SALM group were significantly increased compared to the CON group, STL group, and STH group (*P* = 0.0014, *P* = 0.0070, and *P* = 0.0035) (Fig. 3C), while the liver lysozyme levels were significantly reduced. This confirms that the attack group of broilers suffered severe liver damage. Compared with the CON group, the lysozyme levels in the SALM group of broilers were significantly increased (*P* = 0.0424). After treatment with TA, liver injury was significantly reduced in the STL and STH groups (*P* = 0.0382 and *P* = 0.0030) (Fig. 3D).

### Mitigating effect of TA on *Salmonella*-induced jejunum in broilers

**Fig 4:**
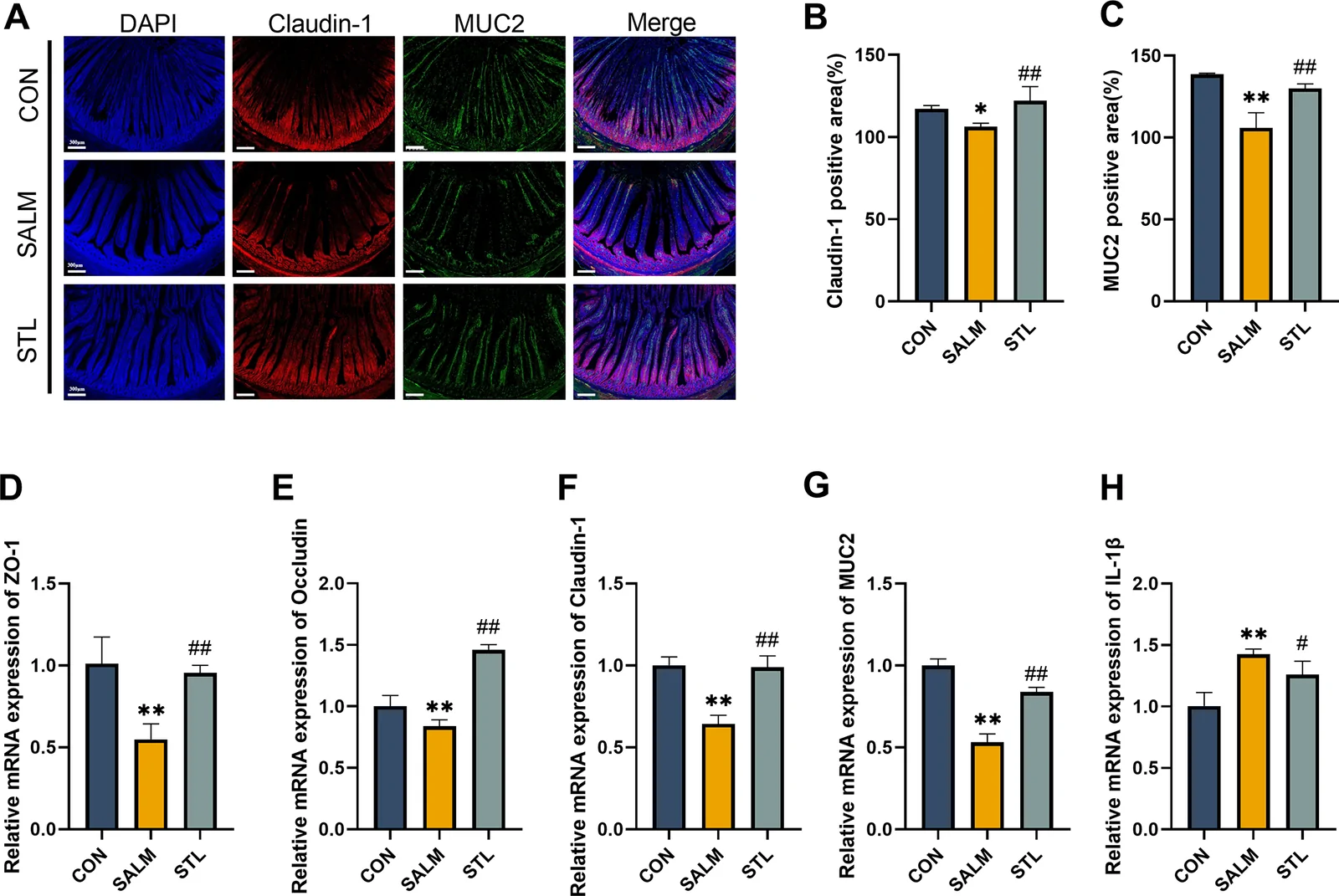
Mitigating effect of tannic acid on *Salmonella*-induced jejunum in broilers. (**A**) Immunofluorescence staining analysis of the expression of Claudin-1 and MUC2 proteins in jejunum tissues (×5; scale bar = 300 µm). (**B**) Results of Claudin-1 immunofluorescence staining image analysis. (**C**) Results of MUC2 immunofluorescence staining image analysis. (**D–H**) Jejunum ZO-1, Occludin, Claudin-1, MUC2, and IL-1β mRNA expression levels; all data are shown as the mean ± SEM (*n* = 5). *n* = 3, all means are analyzed by one-way ANOVA; * denotes the level of significance compared to the CON group, *P < 0.05 and **P < 0.01. # denotes significance compared to the SALM group, #*P* < 0.05 and ##*P* < 0.01.

STL proved more effective in the low-dose TA group based on intestinal and liver phenotypes, so we chose STL for further analysis. We examined intestinal barrier markers to determine if *Salmonella* induction disrupts the barrier and heightens tissue toxicity. As shown in Fig. 4, Occludin, ZO-1, Claudin-1, and MUC2 are intestinal barrier-related markers. The immunofluorescence levels of Claudin-1 (*P* = 0.0161) and MUC2 (*P* < 0.0001) were significantly downregulated in the SALM group compared to the control group. The STL group reversed these two levels of fluorescence immunity (*P* = 0.0012 and *P* < 0.0001, Fig. 4A through C). The levels of ZO-1 (*P* < 0.0001), Occludin (*P* = 0.0039), Claudin-1 (*P* < 0.0001), and MUC2 (*P* < 0.0001) in the SALM group were significantly lower, and the inflammatory factor IL-1β (*P* < 0.0001) mRNA were significantly higher than those in the blank group; however, these levels were reversed in the STL group, consistent with the immunofluorescence results (*P* = 0.0002, *P* < 0.0001, *P* < 0.0001, *P* < 0.0001*,* and *P* = 0.0355) (Fig. 4D through H).

### Effect of TA on *Salmonella*-induced liver immune injury in broilers

**Fig 5:**
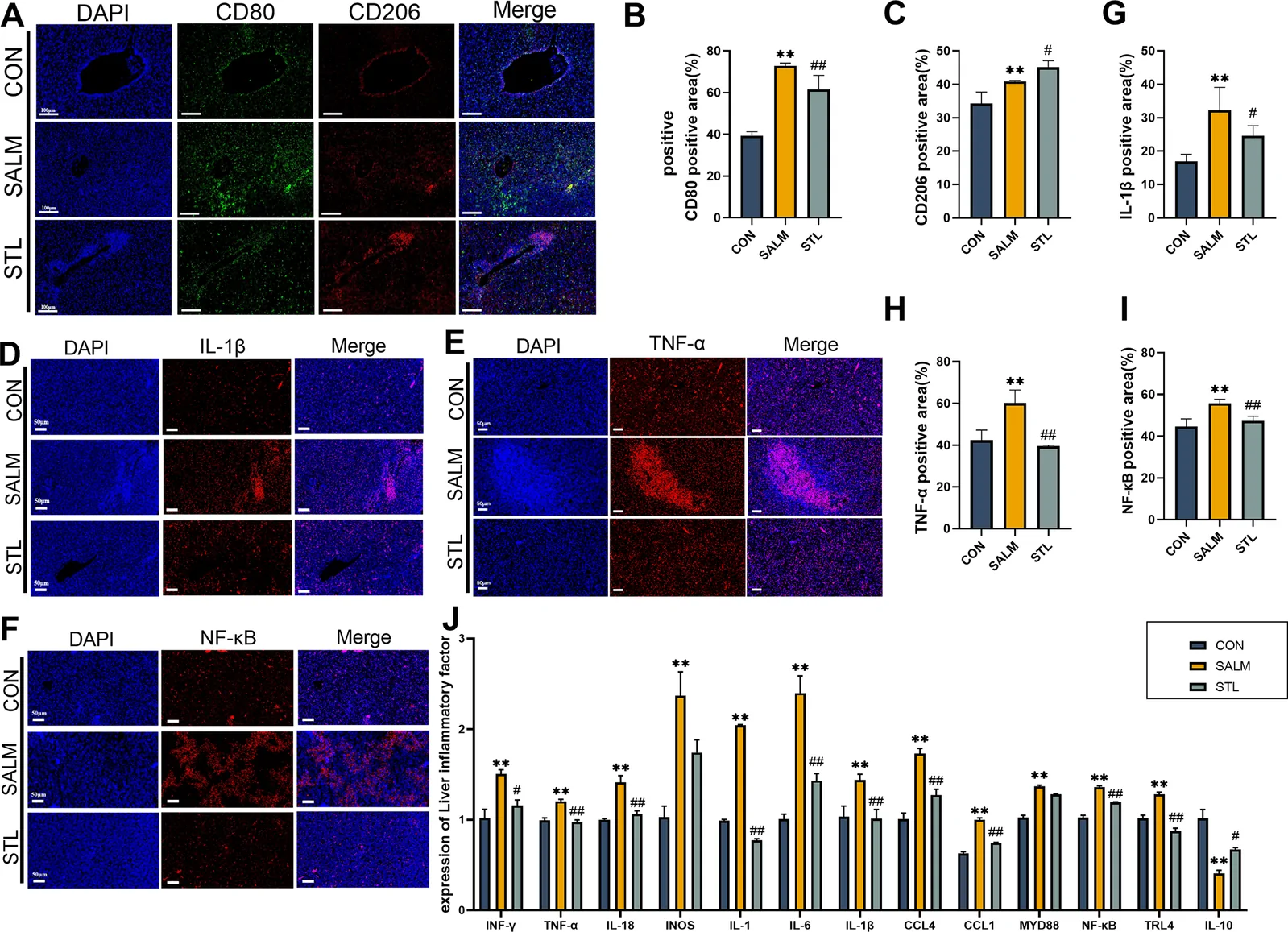
Effects of tannic acid on *Salmonella*-induced immune injury in broiler liver. (**A**) Immunofluorescence staining to analyze CD80 and CD206 protein expression in liver (×20; scale bar = 100 µm). (**B**) Immunofluorescence intensity of CD80 in liver. (**C**) Immunofluorescence intensity of CD206 in liver. (**D–F**) Immunofluorescence staining to analyze IL-1β, TNF-α, and NF-κB protein expression (×20; scale bar = 50 µm). (**G–I**) Liver IL-1β, TNF-α, and NF-κB immunofluorescence intensity. (**J**) Expression levels of IFN-γ, TNF-α, IL-18, INOS, IL-1, IL-6, IL-1β, CCL4, CCL1, MYD88, NF-κB, TRL4, and IL-10 mRNA; all data are shown as the mean ± SEM (*n* = 5). *n* = 3, all means are analyzed by one-way ANOVA; * denotes the level of significance compared to the CON group, *P < 0.05 and **P < 0.01. # denotes significance compared to the SALM group, #*P* < 0.05 and ##*P* < 0.01.

To explore if *Salmonella* liver colonization causes immune injury and if TA can mitigate it, we used immunofluorescence and quantitative real-time PCR (qRT-PCR), as shown in Fig. 5. The immunofluorescence intensity of the M1 Kupffer cell marker CD80 in the SALM group was significantly higher than that in both the CON (*P* < 0.0001) and STL groups (*P* = 0.0026). Conversely, the immunofluorescence intensity of the M2 Kupffer cell marker CD206 in the STL group was significantly elevated compared to the SALM group (*P* = 0.0342) (Fig. 5A through C). Additionally, the liver immunofluorescence results for IL-1β, TNF-α, and NF-κB, which are cytokines associated with inflammation, indicated that fluorescence intensity was significantly higher in the SALM group than in the CON group (*P* = 0.0004, *P* < 0.0001, and *P* = 0.0001). This increase was reversed in the STL group following TA treatment (*P* = 0.046, *P* = 0.001, and *P* < 0.0001) (Fig. 5D through I). For validation, we performed qRT-PCR for hepatic M1- and M2-associated inflammatory cytokines and inflammatory pathway-associated factors. Compared with the CON group, the mRNA expression levels of IFN-γ (*P* = 0.0012), IL-18 (*P* = 0.0001), IL-1β (*P* = 0.0029), IL-1, IL-6, CCL4, CCL1, NF-κB, TNF-α, and TRL4 (all of the above are *P* < 0.0001) were significantly elevated in the SALM group (Fig. 5J). In contrast, these inflammatory factors were significantly reduced in the STL group after TA treatment. Meanwhile, the mRNA level of IL-10 was significantly decreased in the SALM group (Fig. 5J) (*P* < 0.0001). These results indicate that *Salmonella* liver colonization impairs immune function and triggers inflammation, which TA can reduce.

### Effect of TA on *Salmonella*-induced jejunal microbiota in broilers

**Fig 6:**
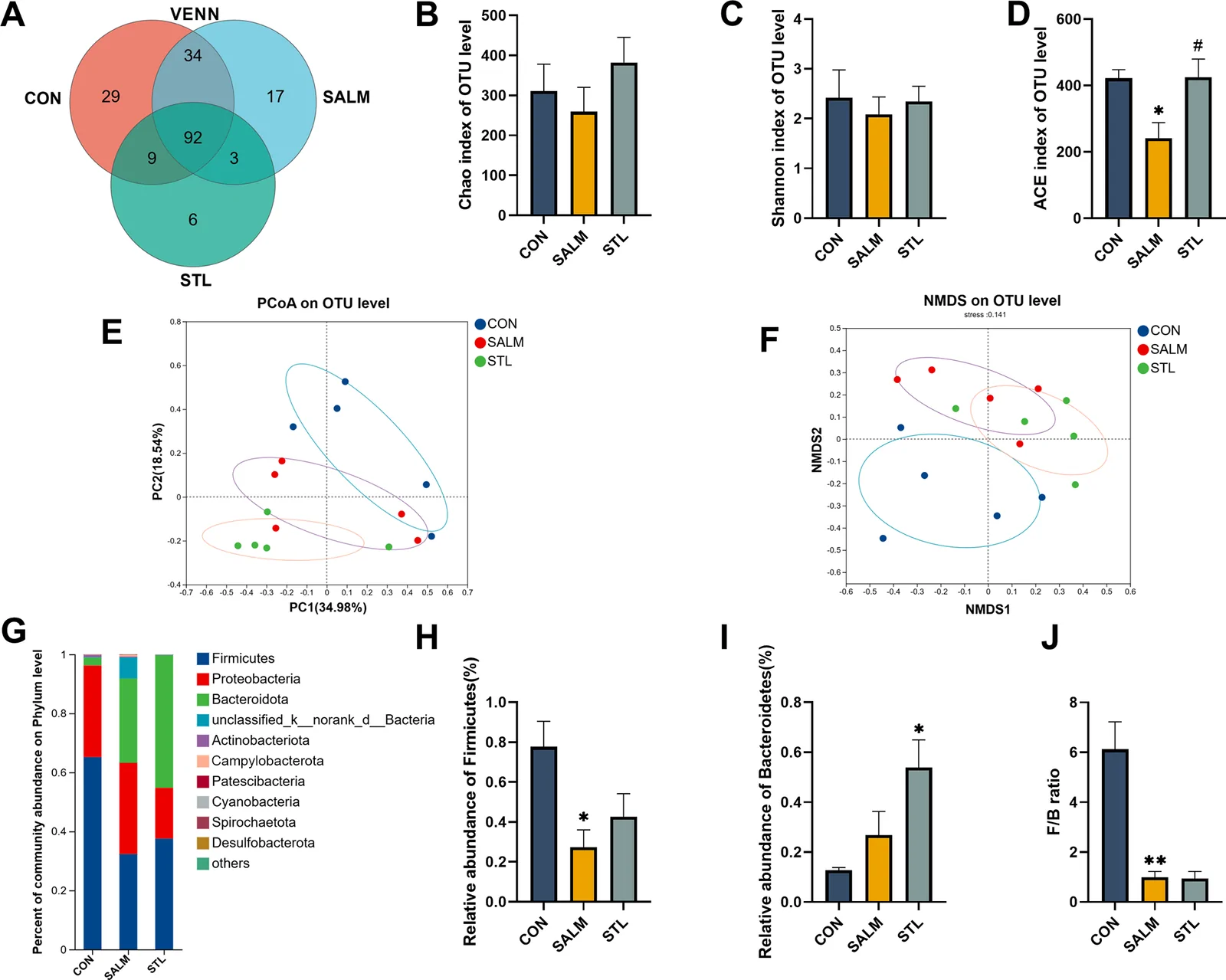
Effect of tannic acid on *Salmonella*-induced jejunal microbiota of broilers. (**A**) VENN plots showing unique and shared OTUs in each group of jejunal microbiota. (**B**) Chao index of OTU. (**C**) Shannon index of OTU. (**D**) ACE index of OTU. (**E**) PCoA of OTU. (**F**) NMDS of OTU. (**G**) Transfer analysis of different phyla in the gut microbiota. (**H**) Abundance levels of *Firmicutes* at the phylum level. (**I**) Abundance levels of *Bacteroidetes* at the phylum level. (**J**) F/B, *Firmicutes*/*Bacteroidetes* ratio; all data are expressed as mean ± SEM (*n* = 5). *n* = 6, all means are analyzed by one-way ANOVA, * denotes the level of significance compared to the CON group, *P < 0.05 and **P < 0.01. # denotes significance compared to the SALM group, #*P* < 0.05 and ##*P* < 0.01.

Furthermore, venn diagrams of operational taxonomic units (OTUs) showed 29 unique OTUs in the CON group, 17 in the SALM group, and 6 in the STL group, with 92 OTUs common to all groups (Fig. 6A). As illustrated in Fig. 6, the analysis of microbial alpha diversity using Chao, Shannon, and ACE indices indicated that the SALM group exhibited significantly lower species richness and diversity compared to the CON (*P* = 0.0332) and STL (*P* = 0.0302) groups (Fig. 6B through D). Changes in gut microbial communities were highlighted by principal coordinate analysis (PCoA) and non-metric multidimensional scaling (NMDS) analysis. The results demonstrated that the gut microbial composition of broilers in the SALM group differed significantly from that of the CON and STL groups (Fig. 6E and F). At the phylum level, the addition of TA altered the abundance of *Firmicutes* and *Bacteroidota* (Fig. 6G). The abundance of *Firmicutes* was higher (*P* > 0.05), and the abundance of *Bacteroidota* was also significantly higher (*P* = 0.0122) compared to the SALM group, while the *Firmicutes*/*Bacteroidetes* (F/B) ratio did not show a significant difference (*P* = 0.004) (Fig. 6H through J).

**Fig 7:**
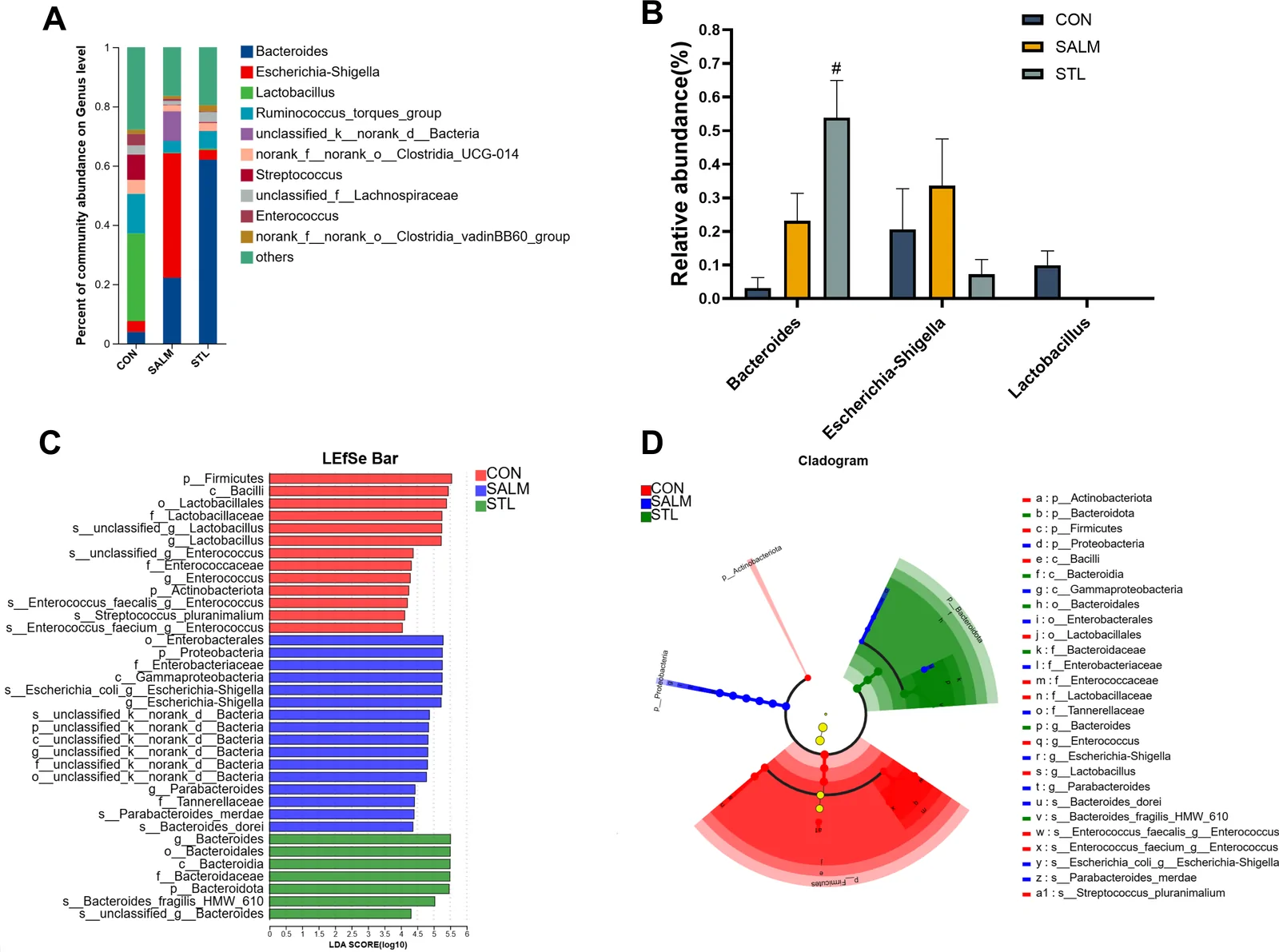
Effect of tannic acid on *Salmonella*-induced jejunal microbiota of broilers. (**A**) Microbiota compositions at the genus level. (**B**) Abundance levels of *Bacteroides*, *Escherichia-Shigella*, and *Lactobacillus* at the genus level. (**C**) Taxonomic cladogram obtained from LEfSe analysis illustrated highly abundant taxa among groups. (**D**) Histogram of LDA displayed the most enriched bacterial taxa in each group (LDA score threshold > 2); *n* = 6, all data are expressed as mean ± SEM (*n* = 5). All means are analyzed by one-way ANOVA, * denotes the level of significance compared to the CON group, *P < 0.05 and **P < 0.01. # denotes significance compared to the SALM group, #*P* < 0.05 and ##*P* < 0.01.

At the genus level, the addition of TA influenced the abundance of *Bacteroides*, *Escherichia-Shigella*, and *Lactobacillus* (Fig. 7A). Further visual analysis indicated that TA significantly increased the relative abundance of the beneficial bacteria *Bacteroides* (*P* < 0.0001). We performed a Pearson correlation analysis to explore the relationship between the jejunal barrier and genus-level bacterial composition in broilers. The linear discriminant analysis effect size (LEfSe) method employed linear discriminant analysis (LDA) to estimate the impact of species abundance on differential effects and identify the bacteria that exhibited these differences. As illustrated in Fig. 7C and D, the LEfSe taxonomic cladogram and histogram of LDA indicated an enrichment of *Bacteroides* in the STL group. *Bacteroides* can enhance immune performance by stimulating the immune system, improving phagocytosis by Kupffer cells, resisting colonization by pathogenic bacteria, and promoting the proliferation of probiotics (28).

**Fig 8:**
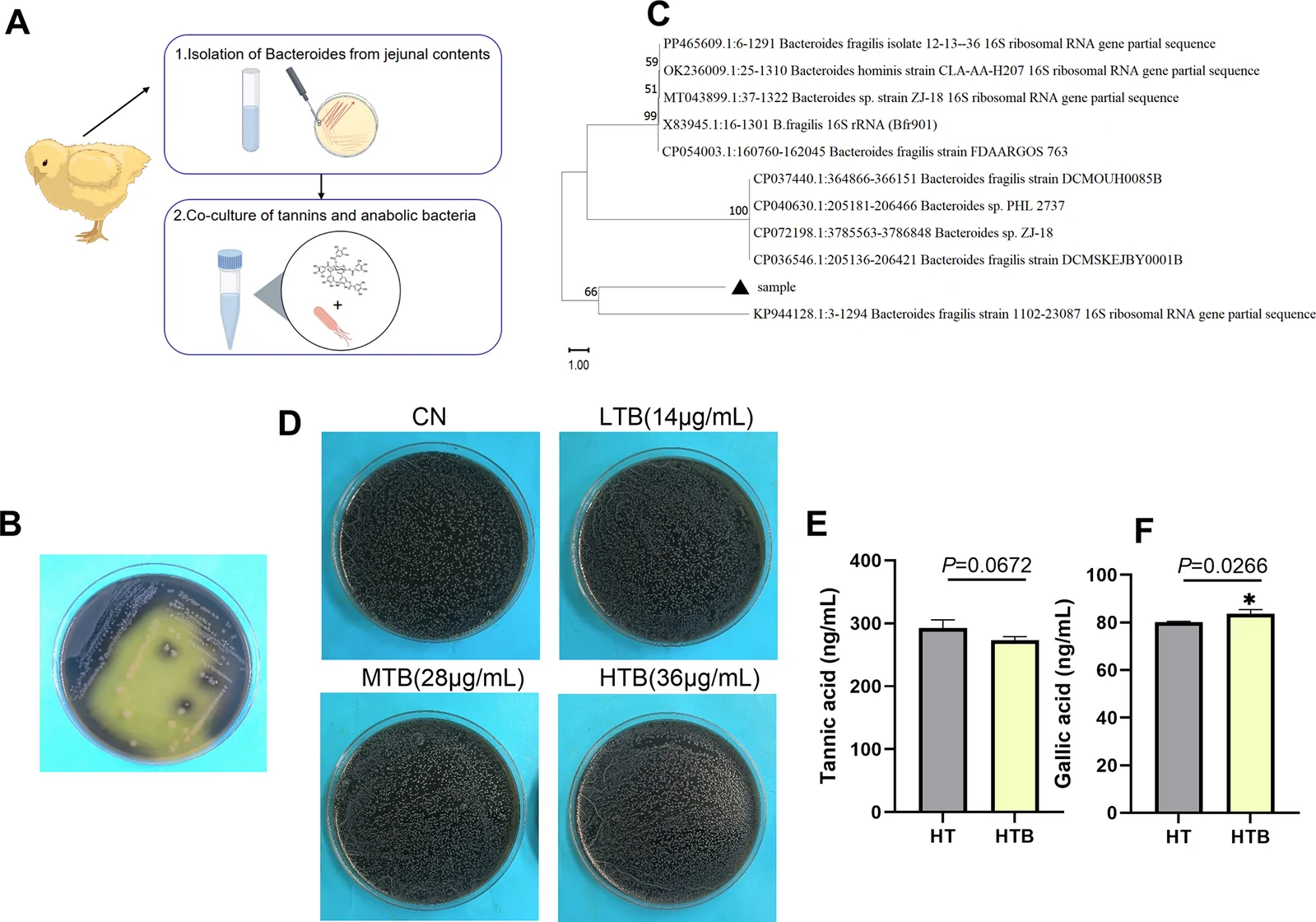
Co-culture system of *Bacteroides* avium and tannic acid. (**A**) Co-culture system flow chart. (**B**) Isolation of *Bacteroides* in BEE medium plates. (**C**) Developmental tree of isolates. (**D**) Growth of *Bacteroides* after treatment with different concentrations of tannic acid. (**E**) Concentration of tannic acid in co-cultured bacterial fluids. (**F**) Concentration of gallic acid in co-cultured bacterial fluids; all data are shown as the mean ± SEM (*n* = 3). *n* = 3, all means are analyzed by one-way ANOVA, * denotes the level of significance compared to the CON group, *P < 0.05 and **P < 0.01. # denotes significance compared to the SALM group, #*P* < 0.05 and ##*P* < 0.01.

We performed a Pearson correlation analysis to explore the relationship between the jejunal barrier and genus-level bacterial composition in broilers. To investigate this, we isolated *Bacteroides* from the intestine for co-culture with TA (Fig. 8A). The target strains were isolated using BEE selection medium (Fig. 8B). The obtained sequencing results of the bacterial strain were analyzed by performing sequence alignment on the NCBI website (https://blast.ncbi.nlm.nih.gov/Blast.cgi). Sequences with the highest homology (≥98%) were retrieved, and a phylogenetic tree was constructed using MEGA11 software. The analysis indicated that the isolated strain shared the highest sequence similarity with *Bacteroides* sp. KP944128 (Fig. 8C). Among the three concentrations of low concentration of tannic acid (LTB, 14 μg/mL), medium concentration of tannic acid (MTB, 28 μg/mL), and high concentration of tannic acid (HTB, 36 μg/mL), we selected the highest concentration of TA for co-culture experiments, as it most effectively promotes the growth of *Bacteroides* (Fig. 8D). After 16–18 hours of co-cultivation, TA showed a non-significant decreasing trend in the co-culture group compared to the blank group (HT) without bacteria (*P* > 0.05) (Fig. 8E). Conversely, the concentration of gallic acid increased significantly (*P* = 0.0266) (Fig. 8F). These findings suggest that *Bacteroides* in the intestine can degrade TA into the more active gallic acid, thereby exerting an antibacterial effect.

### Effect of TA on *Salmonella*-induced liver microbiota in broilers

**Fig 9:**
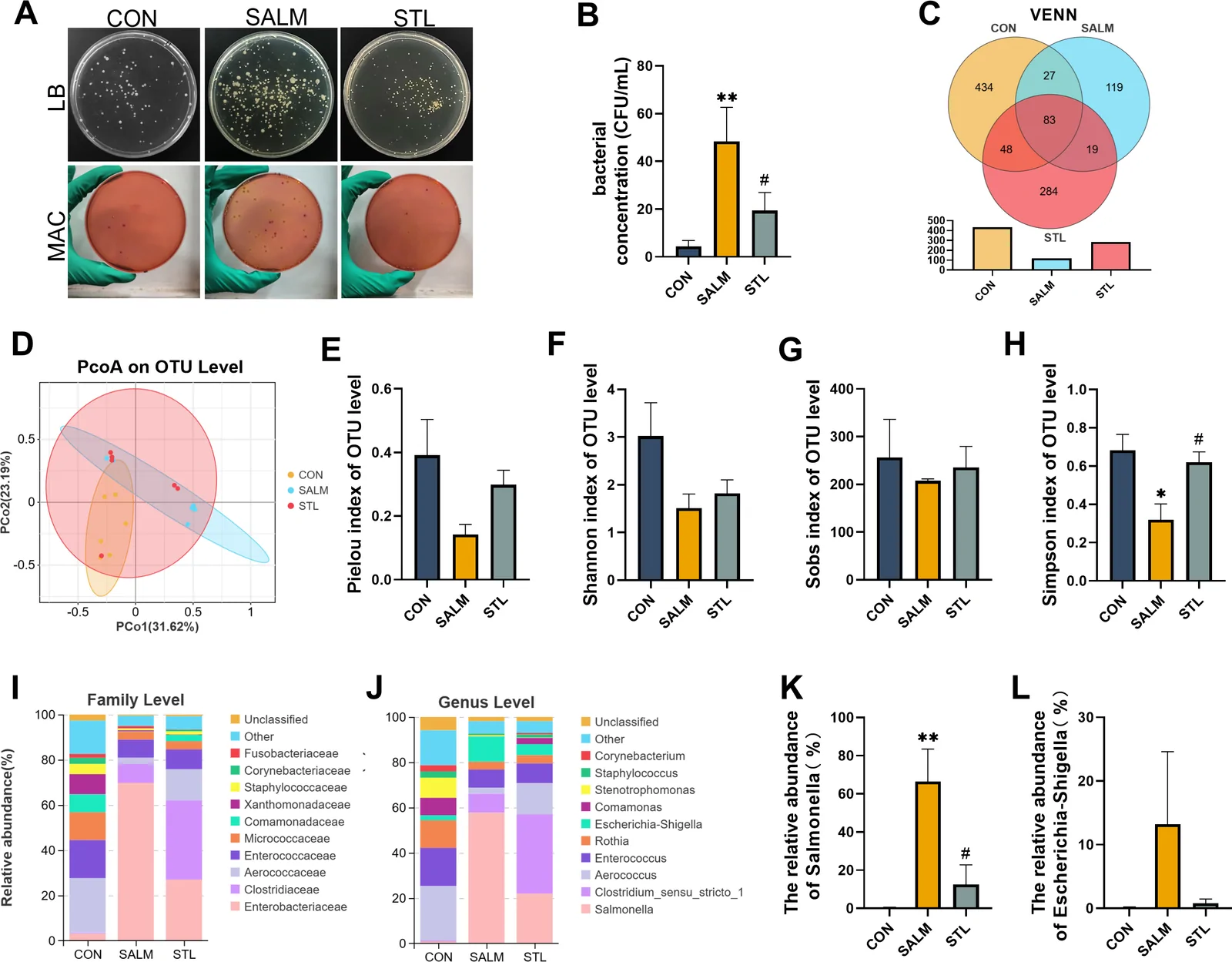
Effect of tannic acid on *Salmonella*-induced microbiota of broiler livers. (**A**) Bacterial culture of liver tissues in LB and MAC media. (**B**) Colony counts in MAC media. (**C**) VENN’s plots showing unique and shared OTUs in each group of liver microbiota. (**D**) PCoA of OTU. (**E**) Pielou index of OTU. (**F**) Shannon index. (**G**) Sobs index of OTU. (**H**) Simpson index of OTU. (**I**) Percentage of abundance of microbiota at the family level. (**J**) Percentage of abundance of microbiota at the genus level. (**K**) Proportion of *Salmonella* spp. in each group. (**L**) Proportion of *Escherichia-Shigella* in each group; all data are shown as the mean ± SEM (*n* = 5). (**A and B**) *n* = 3; (**C–L**) *n* = 6, all means are analyzed by one-way ANOVA, * denotes the level of significance compared to the CON group, *P < 0.05 and **P < 0.01. # denotes significance compared to the SALM group, #*P* < 0.05 and ##*P* < 0.01.

We conducted tissue bacterial cultures and microbiome analyses on liver samples, with the results presented in Fig. 9A and B. Bacterial culture of liver homogenates revealed detectable colony counts across all groups (Fig. 9B). The mean bacterial loads were 4.33, 48.33, and 19.33 CFU/mL for the CON, SALM, and STL groups, respectively. Venn diagrams of operational taxonomic units revealed that 434, 119, and 284 unique OTUs were identified in the CON, SALM, and STL groups, respectively, with a total of 83 OTUs shared among the groups (Fig. 9C). PCoA demonstrated that the microbial composition of liver tissues in CON, SALM, and STL group mice showed certain differences (Fig. 9D). For alpha diversity (*P* = 0.0369), compared to the CON group, the SALM group showed decreasing trends in the Pielou, Shannon, and Sobs indices, with a significant decrease in the Simpson index. In contrast, TA supplementation upregulated the Pielou, Shannon, and Sobs indices and significantly increased the Simpson index (Fig. 9E through H). At the family level, the dominant bacterial groups across all cohorts were *Enterobacteriaceae*, *Clostridiaceae*, and *Aerococcaceae* (Fig. 9I). At the genus level, the predominant taxa were *Salmonella* and *Escherichia-Shigella* (Fig. 9J). Compared to the CON and STL groups, the relative abundance of the genus *Salmonella* was significantly elevated in the SALM group (*P* < 0.05; Fig. 9K), while the increase in *Escherichia-Shigella* was not statistically significant. The addition of TA significantly decreased the abundance of *Salmonella* (*P* = 0.0153) and *Escherichia-Shigella* (*P* > 0.05) (Fig. 9K and L).

**Fig 10:**
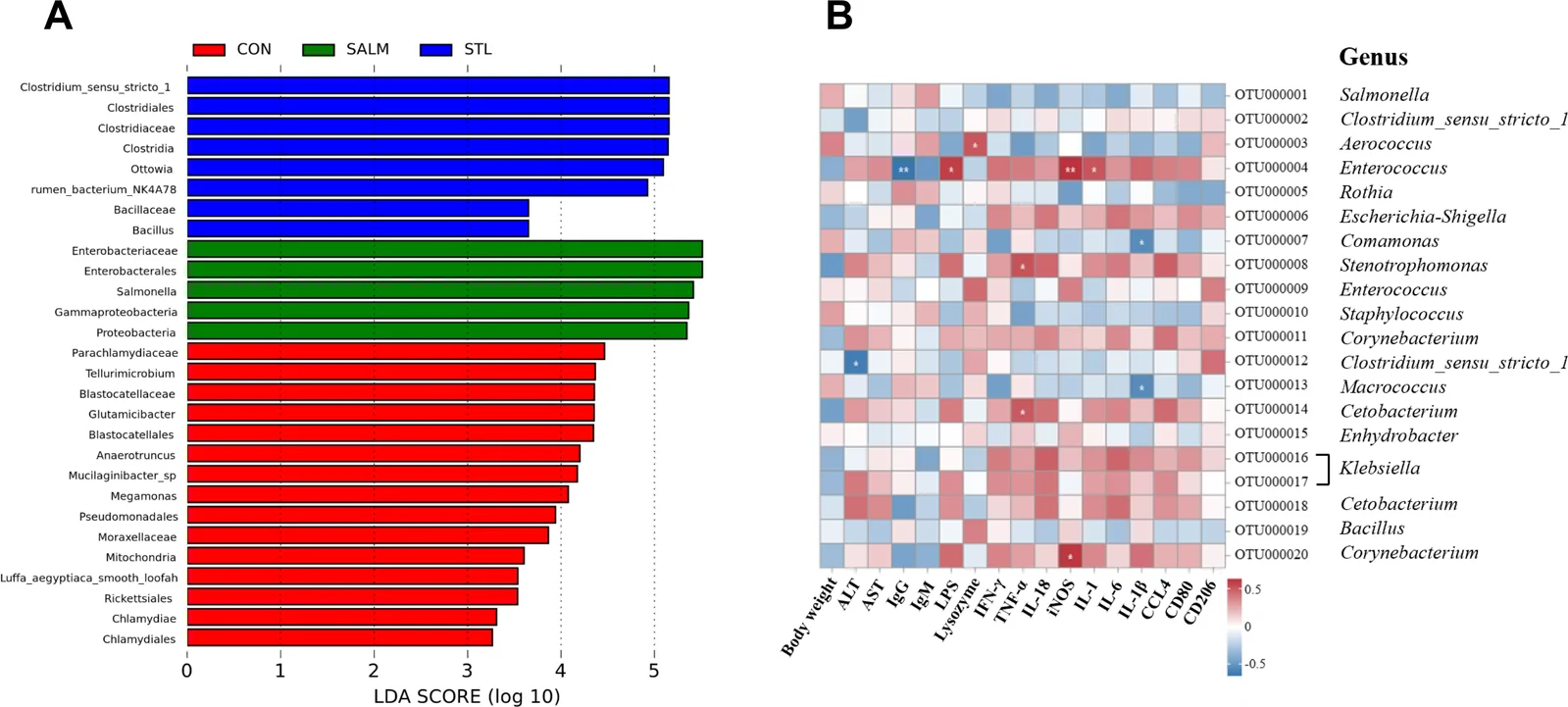
Effect of tannic acid on *Salmonella*-induced microbiota of broiler livers. (**A**) Taxonomic cladogram obtained from LEfSe analysis illustrated highly abundant taxa among groups. (**B**) Heat map of species-environmental factor correlation; all data are shown as the mean ± SEM (*n* = 5). *n* = 6, all means are analyzed by one-way ANOVA, * denotes the level of significance compared to the CON group, *P < 0.05 and **P < 0.01. # denotes significance compared to the SALM group, #*P* < 0.05 and ##*P* < 0.01.

Linear discriminant analysis effect size was applied to the gut microbiota with an LDA threshold of 2 using histograms to identify significantly enriched species in each group, leading to the identification of 28 genera (Fig. 10A). Notably, *Salmonella*, *Enterobacteriaceae*, *Gammaproteobacteria*, and *Proteobacteria* significantly increased in the SALM group, indicating that *Salmonella* can persist in the liver and cause prolonged damage. Subsequently, we analyzed the microbiota of the top 20 most significant genera in relation to hepatic inflammatory factors using random forest analysis (Fig. 10B). A positive correlation was observed between hepatic LPS, iNOS, and IL-1 expression and *Enterococcus* (*P* < 0.05).

## DISCUSSION

The highly contagious nature of salmonellosis enables its swift transmission among poultry populations, potentially resulting in widespread outbreaks. Such pandemics could have serious public health implications and impose significant economic burdens on a global scale (29). Regrettably, contemporary research indicates that *Salmonella* exhibits resistance to multiple antibiotics traditionally employed in its treatment (30). The overuse of antibiotics has precipitated resistance in numerous bacterial strains, thereby engendering a deleterious cycle of escalating antibiotic utilization and bacterial mutation (31, 32). TA, a natural plant extract with various biological activities, could serve as an effective antibiotic alternative. In addition, compared to antibiotics, TA reduces side effects, lowers the risk of resistance development, and is able to increase the potential for overall animal welfare (2). This makes tannins an important candidate for further research in animal health. Compared to antibiotics, TA minimizes side effects, reduces resistance risk, and enhances animal welfare, making tannins a key focus for further animal health research.

Our results indicate that following oral administration of 3 × 10^8^ CFU of *Salmonella* spp., there was a significant decline in the growth performance and intestinal barrier function of broilers. It has been observed that upon reaching the intestinal lumen, *Salmonella* engages with epithelial cells, leading to pathogenic consequences. Subsequently, in the later stages of infection, the bacterium’s interaction with the crypt epithelium induces early inflammatory responses (33). Broilers treated with *Salmonella* showed significantly higher levels of IL-1β and lower levels of MUC2, Claudin-1, ZO-1, and Occludin in comparison to the control group (Fig. 4A). Moreover, intestinal inflammation contributes to the dysbiosis of gut microbiota and the diminution of probiotic species. The inflammatory milieu facilitates the acquisition of nutrients and electron acceptors by *Salmonella*, thereby promoting its proliferation (34, 35). Consistently, there were significant differences in gut microbiota among the groups 10 days after the *Salmonella* challenge. However, *Salmonella* was not detected in the jejunal contents and was detected in the liver after 10 days of *Salmonella* challenge.

Traditionally, the liver is seen as a sterile organ linked to the intestines by the portal vein, which carries nutrient-rich venous blood, microorganisms, and metabolites from the intestines. The portal vein serves as a direct conduit between the intestine and the liver, facilitating the transport of intestinal nutrients and metabolites predominantly to the liver. In instances where the integrity of the intestinal barrier is compromised, this pathway allows for the translocation of harmful bacteria and toxins, such as *Salmonella*, into the bloodstream, which may subsequently reach the liver and cause hepatic damage (36). Under pathological conditions such as *Salmonella* infection, the liver encounters bacterial products like lipopolysaccharide, a key component of gram-negative bacterial cell walls. Our research demonstrated a significant increase in hepatic LPS concentration and *Salmonella* prevalence 10 days post-infection (Fig. 3C and 9K), indicating that both the pathogen and gut bacterial metabolites reach the liver via the gut-liver axis due to barrier impairment (Fig. 4). These translocated components instigate hepatic inflammation, as evidenced by the upregulated expression of Toll-like receptor 4 (TLR-4), MyD88, and NF-κB, which are pivotal mediators of innate immune signaling. The subsequent decrease in pro-inflammatory cytokines (IL-6, IL-18, IL-1β, and TNF-α) is consistent with the known inhibitory effect of TA on NF-κB signaling (Fig. 5D through J). Notably, while intestinal *Salmonella* levels declined over time, the pathogen progressively colonized the liver, indicating active migration through the portal system. This translocation via the gut-liver axis results in sustained hepatic injury and disrupts the liver immune microenvironment, illustrating that *Salmonella* infection leads to both intestinal damage and chronic liver colonization.

This study highlights TA’s dual role in modulating liver immunity and gut microbial balance, as shown by histopathological and 16S rRNA sequencing analyses, underscoring its influence on gut-liver axis regulation. The findings demonstrated that low-dose TA significantly reduced *Salmonella* colonization and mitigated the associated injury and inflammation. It has been documented that TA degrades to gallic acid within the intestine (20), and gallic acid has garnered significant attention due to its potent anti-inflammatory properties (37). In this experiment, the abundance of *Bacteroidota* in the TA treatment group exhibited a significant increase. Furthermore, our findings indicate that *Bacteroides* are capable of metabolizing TA into gallic acid, which possesses anti-inflammatory properties. Gallic acid, a natural antioxidant, effectively reduces inflammation and slows disease progression. Studies show that it can decrease inflammation and oxidative stress in emphysema in mice through the Nrf2-HO-1-NF-κB pathway (38), and it has been shown to ameliorate trinitrobenzene sulfonic acid (TNBS)-induced ulcerative colitis (39). The mitigation of inflammation and the enhancement of the intestinal barrier through TA treatment were substantiated by reduced concentrations of IL-1β and increased levels of MUC2, Claudin-1, ZO-1, and Occludin, as well as immunofluorescence findings in the jejunum of broilers. The *in vitro* conversion of TA requires careful interpretation. The significant increase in gallic acid in the co-culture group demonstrates the metabolic capacity of Bacteroides to transform TA. Notably, the baseline level of gallic acid in the TA-only control suggests possible non-biological degradation during incubation. Thus, the *in vivo* benefits of TA likely stem from a combination of direct bacterial conversion and the bioactive microenvironment shaped by TA and its metabolites. Furthermore, while TA effectively inhibits pathogens, it may also exert a mild, non-selective effect on some commensal bacteria—as evidenced by the lower abundance of *Escherichia-Shigella* in the STL group compared to the CON group. Mechanistically, tannins can inhibit NLRP3 inflammasome-mediated IL-1β production by obstructing NF-κB signaling in macrophages (40). This suppression of NF-κB, in conjunction with its direct bactericidal activity against *Salmonella* (Fig. 5J), synergistically diminishes systemic inflammation through dual antimicrobial and immunomodulatory mechanisms. In comparison to the SALM group, the STL group exhibited minimal traces of *Salmonella* in the liver. This suggests that the fortification of the intestinal barrier by TA potentially hinders the translocation of *Salmonella* to the liver through the gut-liver axis.

The liver functions as a specialized immune organ, distinguished by its dense population of innate immune cells and dual blood supply (portal vein and hepatic artery). This unique architecture exposes it to gut-derived pathogens like *Salmonella*, which translocate following intestinal barrier compromise. Upon hepatic colonization, *Salmonella*-derived LPSs activate TLR-4 on hepatic stellate cells, driving fibrosis and immune injury (41, 42). Simultaneously, macrophages—key regulators of hepatic immunity (43)—undergo phenotypic polarization between proinflammatory M1 and anti-inflammatory M2 states in response to microenvironmental signals (20, 44). Immunofluorescence analysis demonstrated sustained inflammation in *Salmonella*-infected groups, as indicated by increased expression of the M2 marker CD206 and decreased expression of the M1 marker CD80 (Fig. 5A). Importantly, intervention with tannic acid ameliorated this damage through multiple mechanisms: it reduced hepatic M1 Kupffer cell polarization, inhibited NF-κB-mediated inflammatory pathways, and restored metabolic homeostasis. The therapeutic effect of tannic acid was attributed to its dual action: restoration of the intestinal barrier prevented *Salmonella* translocation via the gut-liver axis, while a Bacteroides-enriched microbiota metabolized tannic acid into gallic acid. This bioactive metabolite exerted local anti-inflammatory effects in the gut before reaching the liver, where it resolved inflammation and optimized the immune microenvironment, thereby mitigating *Salmonella*-induced hepatic injury. Notably, the superior efficacy of low-dose TA (200 mg/kg) over the high-dose (400 mg/kg) in our study aligns with the documented biphasic, dose-dependent effects of many plant polyphenols (45). While low doses of polyphenols can exert beneficial effects, high doses may induce metabolic shifts or stress responses that attenuate their efficacy. Therefore, the optimal dosing of TA is crucial to harnessing its protective benefits while avoiding potential adverse outcomes associated with excessive intake.

Our research focuses on the use of tannins as a natural therapeutic agent against *Salmonella*-induced liver injury, compared to traditional antibiotic therapy. Our findings indicate that TA can selectively reduce inflammation and maintain hepatic immune balance without disrupting normal immune function, offering benefits over conventional treatments that might cause immune dysregulation. The decreased *Salmonella* colonization in the jejunum and liver (Fig. 9) has two key food safety benefits: it reduces cross-contamination risks during poultry processing, meeting USDA-FSIS 2023 targets, and supports antibiotic-free poultry production by lowering antimicrobial resistance, thereby decreasing antibiotic-resistant bacteria in the food supply. The application of TA is important for reducing environmental pollution caused by antibiotic use in agriculture and improving the economic sustainability of poultry farming. *In vivo* modeling not only confirms the protective effects of tannins but also shows the potential for this compound to be used in real-world poultry farming practices, providing a practical solution for controlling *Salmonella* infections. Our study concentrated on a single *Salmonella* strain (CICC 21510) and standardized broiler conditions, ensuring internal validity but limiting conclusions about TA’s effectiveness against other *Salmonella* serovars or in real-world co-infections. This may limit the direct applicability of our findings to broader zoonotic threats. Future work will employ zoonotic serovars, such as *Salmonella* Typhimurium, to validate these results. Furthermore, the fixed TA doses (200 and 400 mg/kg feed) may not reflect the variability in commercial feed formulations. While broilers are a key model for poultry farming, more research is necessary to determine if TA consistently affects different species or performs similarly in complex environments. Additionally, more comprehensive toxicity studies are needed to ensure that TA is safe for long-term use in poultry.

### Conclusions

Post*-Salmonella* infection, chicks showed reduced growth and hepatic immune dysregulation due to persistent liver colonization via the gut-liver axis, despite pathogen clearance in the gut. TA demonstrated protective effects against *Salmonella*-induced growth suppression, biochemical dysfunction, and hepatic-jejunal damage in broilers. Mechanistically, TA restored intestinal barrier integrity and microbiota balance, facilitating TA bioconversion to bioactive gallic acid. This metabolite further suppressed hepatic *Salmonella* colonization, attenuated M1 Kupffer cell polarization, inhibited inflammatory cytokine secretion, and ultimately repaired Kupffer cell homeostasis to enhance hepatic immune function.
